# SARS‐CoV‐2 Anti‐S an Anti‐N IgG Seropositivity in Children and Young People (1–24 Years) According to HIV Status in Lomé (Togo) in 2022

**DOI:** 10.1111/irv.70112

**Published:** 2025-04-24

**Authors:** Yao Rodion Konu, Florence Damond, I. Wone Oumarou Adama, Valentine Marie Ferré, Alassane Ouro‐médéli, Ounoo Elom Takassi, Nina Dapam, Magnoulélén N'zonou, Ridwane Bawa‐Kawte, Martin Kouame Tchankoni, Arnold Junior Sadio, Fatoumata Binta Tidiane Diallo, Claver Anoumou Dagnra, Charlotte Charpentier, Didier Koumavi Ekouevi

**Affiliations:** ^1^ Centre de Formation et de Recherche en Santé Publique, Département de Santé Publique, Faculté des Science de la Santé Université de Lomé Lomé Togo; ^2^ GHiGS Team, University of Bordeaux, Inserm (UMR 1219), IRD (EMR 279), Bordeaux Population Health Centre Bordeaux France; ^3^ Service de Virologie, Université Paris Cité, INSERM, IAME, UMR 1137, AP‐HP, Hôpital Bichat‐Claude Bernard Paris France; ^4^ Centre national de recherche sur le VIH, Programme National de Lutte contre le VIH et les hépatites virales Lomé Togo; ^5^ Service de pédiatrie, CHU Sylvanus Olympio Lomé Togo; ^6^ Centre Médicosocial Lucia, ONG Espoir Vie Togo Lomé Togo; ^7^ Service de pédiatrie, Hopital de Bè Lomé Togo; ^8^ Centre médicosocial de l'ONG Action Communautaire pour la Santé Lomé Togo; ^9^ World Health Organization Country Office (WCO) Lomé Togo

**Keywords:** HIV, SARS‐CoV‐2, seropositivity, young people

## Abstract

We aimed to estimate SARS‐CoV‐2 seropositivity among children and young people in Lomé, Togo, according to HIV status. A multicenter comparative cross‐sectional study was conducted, and 636 participants were included (41.8% living with HIV). Anti‐S (88.7% vs. 89.1%) and anti‐N (41.6% vs. 39.5%) IgG seropositivity were comparable in both groups. These data suggest no increased COVID‐19 susceptibility in children and young people with HIV.

## 1 Background

Since the onset of the COVID‐19 pandemic, questions have been raised as to who are at greater risk of COVID‐19 infection and worse outcomes [1]. People living with human immunodeficiency virus (PLWH) were initially suspected, but it remains unclear whether they have an increased susceptibility to SARS‐CoV‐2 infection and severe COVID‐19 manifestations due to HIV‐related immunosuppression [2]. Conversely, it has been suggested that PLWH may be protected against severe manifestations of COVID‐19 because of the initiation of antiretroviral therapy [2].

Understanding whether or not HIV infection could affect susceptibility to SARS‐CoV‐2 infection, its severity, and lower antibody production against SARS‐CoV‐2 is essential both for PLWH and for the healthcare system so as to inform policies and response to future epidemics in which PLWH populations will likely to face [3]. Data on SARS‐CoV‐2 infection and COVID‐19 outcomes in PLWH in Africa is however scarce in general [2]. In particular, little is known about vulnerability faced by children, adolescents, and youths living with HIV to SARS‐CoV‐2 infection.

In sub‐Saharan Africa (SSA) where 66% of PLWH lived in 2023, adolescents and youths aged 10 to 24 years bears the heaviest burden of HIV [4]. The majority (85%) of 1.65 million adolescents living with HIV in the world are found in SSA. Studies have reported poorer HIV care outcomes in adolescents compared to adults including lower viral suppression [5], which could increase the risk of immunosuppression and vulnerability to a range of illnesses, including severe symptoms or death as a result of COVID‐19 [5].

In Togo, seroprevalence of SARS‐CoV‐2 ranged from 50 to 70% in 5‐ to 29‐year‐olds in the general population in 2021 [6]. In this study, we aimed to estimate SARS‐CoV‐2 anti‐S and anti‐N IgG seropositivity among children and young people living with HIV (CYWH) and without HIV (CYWoH) in Lomé (Togo) in 2022.

## 2 Methods

### 2.1 Study Design, Study Sample, and Data Collection

A comparative cross‐sectional study was conducted in four health facilities in Lomé between August and November 2022. The pediatric wards of two tertiary‐level hospitals (Sylvanus OIympio teaching hospital and Bè hosptial) and two care centers for people living with HIV (*Centre Médico‐social Lucia* and *ONG Action Communautaire pour la Santé*) were selected for the study for having a large patient volume. Ethical approval was obtained from the Bioethics Committee for Health Research of the Togo Ministry of Health (N°002/2021/CBRS). Participants meeting the following criteria were included by convenience sampling: i) being 18 months of age or older, ii) seen in consultation or hospitalized during the study period, and iii) given informed consent (and assent for those under 18 years old).

To allow comparison of two proportions, the double population proportion formula was used to estimate a minimum sample size in each group. Assuming an alpha risk of 5%, a beta risk of 20%, a 67.0% seroprevalence in CYWH [6] and 80% seroprevalence in adolescents living with HIV, a minimum of 177 participants per group was thought to be necessary.

A structured questionnaire was administered to child's parent/guardian or participant him/herself (if ≥18 years old) to collect information on sociodemographic characteristics, self‐declared COVID‐19‐related history, and antiretroviral treatment characteristics.

### 2.2 Biological Sample and Analysis

We collected a 5 mL blood sample from each participant. Aliquots of plasma were stored to the laboratory of molecular biology and immunology (BIOLIM, University of Lomé, Togo) and transported frozen to the virology laboratory of the Hôpital Bichat‐Claude Bernard (Paris, France), for the search for anti‐SARS‐CoV‐2 antibodies. SARS‐CoV‐2 anti‐S and anti‐N IgG were measured using the Abbott SARS‐CoV‐2 IgG kits (Alinity i SARS‐CoV‐2 IgG II Quant, and SARS‐CoV‐2 IgG, Abbott, Illinois, United States) with the Alinity i platform according to the manufacturer's instructions (sensitivity > 99% and specificity > 99%) [7].

HIV serology, performed at BIOLIM, was ensured by rapid test (SD Bioline HIV/Syphilis Duo, Abott, Santa Clara, California, United States), and each positive result was confirmed with the First Response® HIV 1–2‐O Card Test (Premier Medical Corporation Pvt. Ltd., Maharashtra, India). In case of discordant results, samples were tested with the INNO‐LIA® HIV I/II Score (20 T) (Fujirebio, Göteborg, Sweden) line immunoassay according to the national guidelines in Togo.

### 2.3 Statistical Analysis

Categorical and quantitative variables were described as proportions and median (with interquartile range [IQR]), respectively. Antibody seroprevalence was estimated with a 95% confidence interval (CI). A linear regression model adjusted for age and sex was fitted to compare anti‐S antibody titer by HIV status in participants positive to SARS‐CoV‐2 anti‐S IgG.

Analyses were carried out using R software version 4.3.2. The predicted means were estimated using the *emmeans* (version 1.11.0) package. The significance level was set at 5%.

## 3 Results

### 3.1 Sociodemographic and HIV‐Related Characteristics

A total of 636 adolescents were included, of which 266 (41.8%) were CYWH. The median age was 17 years (IQR 13–19) in CYWH versus 9 years (IQR 5–14) in CYWoH. Of CYWH, 61.3% were virologically suppressed (viral load <50 copies/mL). The majority (72.6%) has been on antiretroviral therapy for at least 5 years, and 85.7% (*n* = 228/266) were on a dolutegravir‐based regimen (Table 1).

**TABLE 1:** Seroprevalence of SARS‐CoV‐2 anti‐S IgG according to sociodemographic and HIV‐related characteristics (*N* = 636).

Characteristic	HIV negative				HIV positive			
	Overall^a^, *N* = 370^d^	Anti‐S positive^b^, *N* = 330^d^	Anti‐S negative^b^, *N* = 40^d^	*p* value^e^	Overall^a^, *N* = 266^d^	Anti‐S positive^b^, *N* = 236^d^	Anti‐S negative^b^, *N* = 30^d^	*p* value^e^
**Age (years), median (IQR)**	9 (5–14)	9 (5–15)	6 (3–8)	**<0.001**	17 (13–19)	17 (14–20)	12 (9–15)	**<0.001**
**Age (years)**				**0.006**				**<0.001**
<5	96 (25.9)	79 (82.3)	17 (17.7)		2 (0.8)	2 (100.0)	0 (0.0)	
[5–10[	105 (28.4)	91 (86.7)	14 (13.3)		35 (13.2)	22 (62.9)	13 (37.1)	
[10–15[	83 (22.4)	76 (91.6)	7 (8.4)		62 (23.1)	54 (87.1)	8 (12.9)	
[15–20[	42 (11.4)	40 (95.2)	2 (4.8)		109 (41.0)	102 (93.6)	7 (6.4)	
≥20	44 (11.9)	44 (100.0)	0 (0.0)		58 (21.8)	56 (96.6)	2 (3.4)	
**Gender**				**0.008**				0.070
Female	165 (44.7)	155 (93.9)	10 (6.1)		139 (52.3)	128 (92.1)	11 (7.9)	
Male	204 (55.3)	174 (85.3)	30 (14.7)		127 (47.7)	108 (85.0)	19 (15.0)	
Missing data	1	1	0					
**Education level**				0.053				**0.004**
No education	81 (22.0)	69 (85.2)	12 (14.8)		34 (13.2)	33 (97.1)	1 (2.9)	
Primary	161 (43.8)	139 (86.3)	22 (13.7)		74 (28.7)	57 (77.0)	17 (23.0)	
Secondary	66 (17.9)	62 (93.9)	4 (6.1)		118 (45.7)	109 (92.4)	9 (7.6)	
University	60 (16.3)	58 (96.7)	2 (3.3)		32 (12.4)	30 (93.7)	2 (6.3)	
Missing data	2	2	0		8	7	1	
**COVID‐19 symptoms in the last 15 days** ^**c**^								**0.004**
No	134 (36.2)	122 (91.0)	12 (9.0)		213 (79.9)	195 (91.5)	18 (8.5)	
Yes	236 (63.8)	208 (88.1)	28 (11.9)	0.4	53 (20.1)	41 (77.4)	12 (22.6)	
Missing data					2	2	0	
**COVID‐19 vaccination**				**0.016**				0.13
Do not know	26 (7.1)	25 (96.2)	1 (3.8)		1 (0.4)	1 (100.0)	0 (0.0)	
No	289 (78.3)	251 (86.9)	38 (13.1)		236 (89.4)	206 (87.3)	30 (12.7)	
Yes	54 (14.6)	53 (98.1)	1 (1.9)		27 (10.2)	27 (100.0)	0 (0.0)	
Missing data	1	1	0		2	2	0	
**Antiretroviral regimen**								0.7
DTG‐based					228 (85.7)	203 (89.0)	25 (11.0)	
NNRTI‐based					24 (9.0)	20 (83.3)	4 (16.7)	
PI‐based					14 (5.3)	13 (92.9)	1 (7.1)	
**Duration of antiretroviral therapy (years)**								**0.025**
<5					73 (27.4)	58 (79.5)	15 (20.5)	
[5,10[					100 (37.6)	92 (92.0)	8 (8.0)	
[10,15[					73 (27.4)	66 (90.4)	7 (9.6)	
>15					20 (7.5)	20 (100.0)	0 (0.0)	
**Duration of DTG based regimen (month)**								**0.004**
<12					47 (22.3)	37 (78.7)	10 (21.3)	
[12,24[					92 (43.6)	82 (89.1)	10 (10.9)	
>24					72 (34.1)	70 (97.2)	2 (2.8)	
Missing data					55	47	8	
**HIV viral suppression (<50 c/mL)**					163 (61.3)	145 (89.0)	18 (11.0)	0.9
**HIV viral load <200 c/mL**					200 (75.2)	178 (89.0)	22 (11.0)	0.8
**HIV viral load <1000 c/mL**					218 (81.9)	195 (89.4)	23 (10.6)	0.4

Abbreviations: IQR, interquartile range; DTG, dolutegravir.

^a^ Proportions were calculated by dividing the modality number by the column total.

^b^ Proportions are calculated by dividing the number of modalities by the total for the line (anti‐S positive + anti‐S negative).

^c^ At least one of the following symptoms: fever, headache, joint/muscle pain, sore throat, rhinorrhea, shortness of breath, ageusia, anosmia, abdominal pain, diarrhea, cough, and unusual fatigue.

^d^ Median (25–75%); *n* (%).

^e^ Wilcoxon rank sum test; Fisher's exact test; Pearson's chi‐squared test.

### 3.2 COVID‐19 Related History

Of 266 CYWH, 20.1% (*n* = 53) reported having had at least one symptom of COVID‐19 in the 15 days prior to inclusion, and 10.2% (*n* = 27) reported having been vaccinated against COVID‐19. These proportions were respectively 63.8% (*n* = 236/370) and 14.6% (*n* = 54/370) among CYWoH (Table 1).

### 3.3 SARS‐CoV‐2 IgG Seropositivity

Overall, seropositivity of SARS‐CoV‐2 anti‐S IgG was 89.0% (566/636). Among CYWH, seropositivity was 88.7% (95%CI 84.1–92.1), compared with 89.1% (95%CI 85.5–92.1) CYWoH (Table 1). Among participants who reported not having been vaccinated against COVID‐19, seropositivity was 87.3% among CYWH versus 87.6% CYWoH (Supplementary Table S1).

In the multiple linear regression model, CYWH had on average 108 BAU/mL fewer anti‐S antibody titers (β = −108, 95%CI −184, −31) adjusted for age and gender (Supplementary Table S2).

Finally, 40.7% (*n* = 259/636) participants where positive to SARS‐CoV‐2 anti‐N IgG. This seropositivity was 41.6% (95%CI 36.6–46.8) among CYWH, compared with 39.5% (95%CI 33.6–45.6) among CYWoH (Supplementary Table S3).

## 4 Discussion

This study was carried out between August and November 2022, more than 2 years after the first COVID‐19 case was detected in Togo. We observed that SARS‐CoV‐2 anti‐S and anti‐N seropositivity was comparable between CYWH and CYWoH. In a repeat SARS‐CoV‐2 seroprevalence survey amongst CALWHIV in Europe (*n* = 493) and South Africa (SA, *n* = 307), and HIV‐negative adolescents in South Africa (*n* = 100), in 2020–2022, Jackson et al., questioned the susceptibility of children and adolescents living with HIV to SARS‐CoV‐2. They reported that SARS‐CoV‐2 seroprevalence was 55% (50–59%) in participant with HIV in Europe, 67% (61–72%) in SA, and 85% (77–92%) among HIV negative participants in SA [8]. Similarly, in a study of 6 cohorts from 55,349 PLWH to over 3.7 million HIV‐free subjects, the proportion of patients testing positive for SARS‐CoV‐2 was comparable according to HIV status [9]. In Uganda, a lower proportion of anti‐S antibodies was reported in PLWH (58.8% vs. 84.9%, *p* = 0.009), while anti‐N antibodies were comparable (68.2% vs. 60.0%, *p* = 0.344) [10]. All this reinforces the idea of a lack of association between HIV infection and susceptibility to SARS‐CoV‐2 infection in children and young people [8], as well as demonstrated in adult populations [11].

In general, anti‐N persists less longer than anti‐S [10], which can explain the results observed with a lower proportion of participants with anti‐N positivity. Elsewhere, the proportion of anti‐S can be modulated by vaccination. Nevertheless, the results observed in our study remain high in a sample in which only 12.8% reported having been vaccinated. We therefore argue that vaccination rates did not influence our results.

Among anti‐S positive participants, HIV positive ones had lower anti‐S titers than their counterparts. Similar findings were made by Spinelli in adult sample [3]. Several studies reported that accumulation of mutations leading to hypermutated variants of SARS‐CoV‐2 may appear during these persistent cases of COVID‐19 [12]. These observations might suggest that PLWH, especially those with immunosuppression, should be prioritized for COVID‐19 risk reduction, including vaccination [12]. This could prevent the emergence of mutations. However, in the absence of an anti‐S titer cutoff this should be further investigated.

This study has several limitations. Firstly, the study sample was drawn using a convenience sampling, which increases the risk of selection bias. Secondly, in the absence of computerized individual medical records, data on the history of COVID‐19 symptoms and vaccination history were collected on the basis of self‐reports. A reporting bias cannot therefore be excluded. Thirdly, anti‐S IgG titers among the two groups of participants should be considered with caution, since there was no SARS‐CoV‐2 PCR available and no data regarding clinical symptoms older than 15 days.

Taking these limitations into account, the results of this study remain relevant, as to our knowledge, it is one of the few investigations exploring the susceptibility of CYWH to SARS‐CoV‐2 infection in sub‐Saharan Africa in comparison with CYWoH. These initial data suggest no increased COVID‐19 susceptibility in CYWH. In the longer term, it would be useful for HIV managing agencies to consider a surveillance strategy for this population in the context of emerging epidemics, in order to identify specific risks and subsequently bring adequate responses.

## Author Contributions

Y.R.K., F.B.T.D., C.C., C.A.D., and D.K.E. conceived, designed, and developed the protocol. I.W.O.A., O.E.T., N.D., M.N., and R.B.K. collected the data. A.O.M., F.D., and V.M.F. performed lab procedures. Y.R.K., M.K.T., and A.J.S. managed and analyzed the data. Y.K.R. and I.W.O.A. drafted the manuscript. All authors reviewed the manuscript.

## Ethics Statement

This study was approved by the Bioethics Committee for Health Research of the Ministry of Health (Number 002/2021/CBRS).

## Consent

Informed consent was provided by parents/caregivers and assent was given by children/adolescents.

## Conflicts of Interest

The authors declare no conflicts of interest.

### Peer Review

The peer review history for this article is available at https://www.webofscience.com/api/gateway/wos/peer‐review/10.1111/irv.70112.

## Permission to Reproduce Material From Other Sources

Not applicable.

## Supporting information


**Table S1** Seroprevalence of anti‐N IgG and anti‐S IgG in participants not vaccinated against COVID‐19 (*N* = 552).
**Table S2** Factors associated with the titer of anti‐S antibodies in seropositive individuals based on a linear regression model (*N* = 566).
**Table S3** Seroprevalence of SARS‐CoV‐2 anti‐N IgG according to sociodemographic and HIV‐related characteristics (*N* = 636).

## Data Availability

Data may be obtained from a third party and are not publicly available. The data are obtainable from the Ministry of Health in Togo.
