# Supplementary material for: SARS‐CoV‐2 Anti‐S an Anti‐N IgG Seropositivity in Children and Young People (1–24 Years) According to HIV Status in Lomé (Togo) in 2022
Source: Influenza Other Respir Viruses. 2025 Apr 24;19(4):e70112. doi: 10.1111/irv.70112 (PMC12022004; doi:10.1111/irv.70112)
Supplement: Supplementary file 1 — Table S1 Seroprevalence of anti‐N IgG and anti‐S IgG in participants not vaccinated against COVID‐19 (N = 552). Table S2 Factors associated with the titer of anti‐S antibodies in seropositive individuals based on a linear regression model (N = 566). Table S3 Seroprevalence of SARS‐CoV‐2 anti‐N IgG according to sociodemographic and HIV‐related characteristics (N = 636). [file IRV-19-e70112-s001.docx]

**Supplementary table 1.** Seroprevalence of anti-N IgG and anti-S IgG in participants not vaccinated against COVID-19 (N=552)

| **Characteristic** | **HIV positive**  N = 237 | **HIV negative**  N = 315 | **Overall**  N = 552 | **p-value** |
| --- | --- | --- | --- | --- |
| **Anti-N IgG seropositivity, n (%)** |  |  |  | **0.010^1^** |
| Positive | 139 (58.6) | 218 (69.2) | 357 (64.7) |  |
| Negative | 98 (41.4) | 97 (30.8) | 195 (35.3) |  |
| **Anti-S IgG seropositivity, n (%)** |  |  |  | 0.922^1^ |
| Positive | 207 (87.3) | 276 (87.6) | 483 (87.5) |  |
| Negative | 30 (12.7) | 39 (12.4) | 69 (12.5) |  |
| **Anti-S IgG concentration (BAU/mL), mean (SD)** | 214 (315) | 227 (377) | 222 (352) | 0.651^2^ |
| ^1^Pearson's Chi-squared test  ^2^Welch Two Sample t-test | | | | |
|  | | | | |

**Supplementary table 2.** Factors associated with the titer of anti-S antibodies in seropositive individuals based on a linear regression model (N=566)

| **Characteristic** | **Estimated marginal average** | |  | **Multiple linear regression** | | |
| --- | --- | --- | --- | --- | --- | --- |
|  | **Value** | **95% CI^1^** |  | **Beta** | **95% CI** | **p-value** |
| (Intercept) |  |  |  | 159 | 74, 244 | <0.001 |
| HIV Status |  |  |  |  |  |  |
| HIV - | 338 | 292, 385 |  | — | — |  |
| HIV + | 231 | 175, 287 |  | -108 | -184, -31 | 0.006 |
| Age (years) | 285 | 250, 319 |  | 13 | 6.9, 19 | <0.001 |
| Sex |  |  |  |  |  |  |
| Female | 271 | 223, 319 |  | — | — |  |
| Male | 298 | 250, 346 |  | 27 | -40, 95 | 0.4 |
| 1 CI = Confidence Interval |  |  |  |  |  |  |

**Supplementary table 3.** Seroprevalence of SARS-CoV-2 anti-N IgG according to sociodemographic and HIV-related characteristics (N=636)

|  | **HIV Negative** | | | |  | **HIV Positive** | | | |
| --- | --- | --- | --- | --- | --- | --- | --- | --- | --- |
| **Characteristic** | **Overall**  N = 370^1^ | **Anti-N**  **Negative**  N = 216^1^ | **Anti-N**  **Positive**  N = 154^1^ | **p-value**^2^ | **Overall**  N = 266^1^ | **Anti-N**  **Negative**  N = 161^1^ | **Anti-N**  **Positive**  N = 105^1^ | **p-value**^2^ |  |
| **Age (years), median (IQR)** | 9 (5-14) | 9 (5-14) | 9 (5-15) | 0.7 | 17 (13-19) | 17 (13-19) | 16 (13-20) | 0.7 |  |
| **Age (years)** |  |  |  | 0.4 |  |  |  | 0.7 |  |
| <5 | 96 (25.9) | 56 (58.3) | 40 (41.7) |  | 2 (0.8) | 2 (100.0) | 0 (0.0) |  |  |
| [5-10[ | 105 (28.4) | 61 (58.1) | 44 (41.9) |  | 35 (13.2) | 23 (65.7) | 12 (34.3) |  |  |
| [10-15[ | 83 (22.4) | 53 (63.9) | 30 (36.1) |  | 62 (23.3) | 35 (56.5) | 27 (43.5) |  |  |
| [15-20[ | 42 (11.4) | 19 (45.2) | 23 (54.8) |  | 109 (41.0) | 68 (62.4) | 41 (37.6) |  |  |
| >=20 | 44 (11.9) | 27 (61.4) | 17 (38.6) |  | 58 (21.8) | 33 (56.9) | 25 (43.1) |  |  |
| **Sex** |  |  |  | >0.9 |  |  |  | 0.4 |  |
| Female | 165 (44.7) | 96 (58.2) | 69 (41.8) |  | 139 (52.3) | 81 (58.3) | 58 (41.7) |  |  |
| Male | 204 (55.3) | 119 (58.3) | 85 (41.7) |  | 127 (47.7) | 80 (63.0) | 47 (37.0) |  |  |
| (Missing data) | 1 | 1 | 0 |  |  |  |  |  |  |
| **Education level** |  |  |  | 0.8 |  |  |  | 0.9 |  |
| No education | 81 (22.0) | 45 (55.6) | 36 (44.4) |  | 34 (13.2) | 22 (64.7) | 12 (35.3) |  |  |
| Primary | 161 (43.8) | 99 (61.5) | 62 (38.5) |  | 74 (28.7) | 42 (56.8) | 32 (43.2) |  |  |
| Secondary | 66 (17.9) | 37 (56.1) | 29 (43.9) |  | 118 (45.7) | 71 (60.2) | 47 (39.8) |  |  |
| University | 60 (16.3) | 34 (56.7) | 26 (43.3) |  | 32 (12.4) | 18 (56.3) | 14 (43.8) |  |  |
| (Missing data) | 2 | 1 | 1 |  | 8 | 8 | 0 |  |  |
| **COVID-19 symptoms on the last 15 days^£^** | 236 (63.8) | 141 (59.7) | 95 (40.3) | 0.5 | 53 (20.1) | 41 (77.4) | 12 (22.6) | **0.005** |  |
| (Missing data) |  |  |  |  | 2 | 1 | 1 |  |  |
| **COVID-19 vaccination** |  |  |  | 0.3 |  |  |  | >0.9 |  |
| Don’t know | 26 (7.0) | 17 (65.4) | 9 (34.6) |  | 1 (0.4) | 1 (100.0) | 0 (0.0) |  |  |
| No | 289 (78.4) | 172 (59.5) | 117 (40.5) |  | 236 (89.4) | 144 (61.0) | 92 (39.0) |  |  |
| Yes | 54 (14.6) | 27 (50.0) | 27 (50.0) |  | 27 (10.2) | 16 (59.3) | 11 (40.7) |  |  |
| (Missing data) | 1 | 0 | 1 |  | 2 | 0 | 2 |  |  |
| **Antiretroviral regimen** |  |  |  |  |  |  |  | 0.2 |  |
| DTG based |  |  |  |  | 228 (85.7) | 138 (60.5) | 90 (39.5) |  |  |
| NNRTI based |  |  |  |  | 24 (9.0) | 17 (70.8) | 7 (29.2) |  |  |
| PI based |  |  |  |  | 14 (5.3) | 6 (42.9) | 8 (57.1) |  |  |
| **Duration of antiretroviral therapy (years)** |  |  |  |  |  |  |  | 0.079 |  |
| <5 |  |  |  |  | 73 (27.3) | 50 (68.5) | 23 (31.5) |  |  |
| [5,10) |  |  |  |  | 100 (37.6) | 56 (56.0) | 44 (44.0) |  |  |
| [10,15) |  |  |  |  | 73 (27.4) | 47 (64.4) | 26 (35.6) |  |  |
| ≥15 |  |  |  |  | 20 (7.5) | 8 (40.0) | 12 (60.0) |  |  |
| **Duration of DTG based regimen (month)** |  |  |  |  |  |  |  | **0.004** |  |
| <12 |  |  |  |  | 47 (22.3) | 38 (80.9) | 9 (19.1) |  |  |
| [12,24) |  |  |  |  | 92 (43.6) | 53 (57.6) | 39 (42.4) |  |  |
| ≥ 24 |  |  |  |  | 72 (34.1) | 37 (51.4) | 35 (48.6) |  |  |
| (Missing data) |  |  |  |  | 55 | 33 | 22 |  |  |
| **HIV viral load <50 c/mL** |  |  |  |  | 163 (61.3) | 88 (54.0) | 75 (46.0) | **0.006** |  |
| **HIV viral load <200 c/mL** |  |  |  |  | 200 (75.2) | 113 (56.5) | 87 (43.5) | **0.019** |  |
| **HIV viral load <1000 c/mL** |  |  |  |  | 218 (82.0) | 124 (56.9) | 94 (43.1) | **0.010** |  |
| ^1^Median (25%-75%); n (%) | | | | | | | | | |
| ^2^Wilcoxon rank sum test; Fisher's exact test; Pearson's Chi-squared test  £ At least one of the following symptoms : Fever, Headache, Joint/muscle pain, Sore throat, Rhinorrhea, Shortness of breath, Ageusia, Anosmia, Abdominal pain, Diarrhea, Cough and Unusual fatigue | | | | | | | | | |
